# Construction of Circulating MicroRNAs-Based Non-invasive Prediction Models of Recurrent Implantation Failure by Network Analysis

**DOI:** 10.3389/fgene.2021.712150

**Published:** 2021-07-23

**Authors:** Peigen Chen, Tingting Li, Yingchun Guo, Lei Jia, Yanfang Wang, Cong Fang

**Affiliations:** Reproductive Medicine Center, The Sixth Affiliated Hospital, Sun Yat-sen University, Guangzhou, China

**Keywords:** recurrent implantation failure, competing endogenous RNAs, assisted reproductive technology, GEO, non-invasive prediction model

## Abstract

**Background:**

Recurrent implantation failure (RIF) is an obstacle in the process of assisted reproductive technology (ART). At present, there is limited research on its pathogenesis, diagnosis, and treatment methods.

**Methods and Results:**

In this study, a series of analytical tools were used to analyze differences in miRNAs, mRNAs, and lncRNAs in the endometrium of patients in a RIF group and a control group. Then the competing endogenous RNA (ceRNA) network was built to describe the relationship between gene regulation in the endometrium of the RIF group. Based on the results of the logistic regression of co-expression miRNAs between serum and endometrial samples, we built a predictive model based on circulating miRNAs.

**Conclusion:**

The stability and non-invasiveness of the circular miRNA prediction model provided a new method for diagnosis in RIF patients.

## Introduction

Recurrent implantation failure (RIF) is a thorny issue that couples undergoing *in vitro* fertilization (IVF)/intracytoplasmic sperm injection (ICSI) may face. The generally accepted definition is that women under the age of 40 years have transferred at least four high-quality embryos in at least three fresh or frozen cycles or have transferred a total of 10 high-quality embryos but have not yet achieved clinical pregnancy (Thornhill et al., 2005; Simon and Laufer, 2012; Coughlan et al., 2014). Along with improving *in vitro* fertilization embryo transfer (IVF-ET) technology and increasing clinical pregnancy rate, RIF is still a tough problem in the process of IVF-ET. The normal embryo implantation generally only occurs during the window of implantation (WOI) (Cha et al., 2012), which refers to days 20–24 of the normal menstrual cycle. Abnormalities of the endometrium at this stage are important factors that lead to RIF.

MicroRNAs (miRNA) are a class of non-coding RNA molecules with a length of about 22 nucleotides that are widely found in eukaryotic cells. There have been some studies confirming the role of miRNA in endometrial regulation (Creighton et al., 2010; Kuokkanen et al., 2010;

Revel et al., 2011; Altmae et al., 2013). For example, miR-30b, miR-30d, and miR-494 had been reported to play an important role in the regulation of endometrial function (Altmae et al., 2013). Recent research reported miRNAs associated with RIF, such as miR-34c-5p (Tan et al., 2020) and miR-148A-3P (Zhang et al., 2020).

Moreover, in recent years circulating miRNA has been increasingly used as a non-invasive tool for disease diagnosis and prediction due to its high stability, sensitivity, and specificity (Martinez and Peplow, 2020). In the present study, we aim to use a larger sample size of data in our analysis to explore the regulatory molecular mechanism in the endometrium of RIF patients at the WOI stage. At the same time, we aim to look for peripheral blood miRNAs closely related to RIF and provide a new way for non-invasive early diagnosis of RIF, thereby improving the clinical outcome of patients.

## Materials and Methods

We used R software (version 3.6.3) (Team, 2018), GraphPad Prism (version 8), and Bioconductor (Gentleman et al., 2004) for all statistical analyses in our study.

### Data Acquisition and Preprocessing

Paired serum and endometrial miRNA expression profile data (GSE108966) were obtained from the Gene Expression Omnibus (GEO) database^1^. The paired raw count of endometrial expression profile and corresponding clinical data of a RIF group and a control group were extracted from GSE71331 and GSE71332 and then processed by “Limma” R package (Ritchie et al., 2015) (Agilent-052909 CBC lncRNA mRNA V3, Agilent-046064 Unrestricted Human miRNA V19.0).

### Selection of Differentially Expressed Genes

The scanning of differentially expressed (DE) miRNA in the endometrium and the serum was performed by using the “limma” R package (Ritchie et al., 2015) with the following criteria: *p*-value < 0.05 and | log 2-fold change| > 1.

Similar to the above process, the differentially expressed genes (DEGs) of GSE71331 and GSE71332 were selected.

### Selection and Validation of Co-expression miRNAs Between Serum and Endometrial Samples

The intersection of endometrium DE miRNAs and serum DE miRNAs was taken as intersection miRNAs. To ensure that their expressions were relevant, Pearson correlation analysis is performed by using GraphPad Prism (version 8). Genes with the Pearson correlation coefficient | r| ≥ 0.5 were considered to be co-expressed miRNAs between the serum and the endometrium.

### Weighted Correlation Network Analysis of miRNA of Endometrial Samples

With the “WGCNA” R package (Langfelder and Horvath, 2008), weighted correlation network analysis (WGCNA) was performed on DE miRNAs which were selected based on GSE71332 dataset. The minimum gene dendrogram size of average linkage hierarchical clustering was set as 40. Then the dissimilarity and constructed module dendrograms of these modules were calculated.

To estimate the significance of each module and also measure the relationships between genes and sample traits, the gene significance (GS) of each module was then calculated. The GS and module membership (MM, the correlation between the genes in the module and their expression profiles) of every key gene were calculated with the following thresholds: correlation gene GS > 0.5 and correlation gene MM > 0.8.

### Prediction of Target lncRNAs/mRNAs of RIF-Related DE miRNAs

The intersection of the DEGs and the genes of the key modules related to RIF in WGCNA was taken as RIF-related DE miRNAs. Then miRDB^2^ (Chen and Wang, 2020), miRTarBase^3^ (Hsu et al., 2011), and TargetScan^4^ (Agarwal et al., 2015) were used to predict miRNA-targeting mRNAs. NPInter^5^ (Teng et al., 2020) and DIANA-LncBase (Paraskevopoulou et al., 2016) were used to predict miRNA-targeting lncRNAs. The intersection of differential mRNAs/lncRNAs in GSE71331 and the miRNA-targeting mRNAs/lncRNAs were taken as targeting-DE mRNAs/lncRNAs.

### Construction of lncRNA-miRNA-mRNA Regulatory Network

LncRNA-miRNA interactomes were then built based on targeting-DE lncRNAs and RIF-related DE miRNAs. Similarly, mRNA-miRNA interactomes were built. Subsequently, lncRNA--miRNA--mRNA regulatory networks were constructed by using cytoscape, version 3.8^6^. Key modules were selected by MCODE using default parameters (Bader and Hogue, 2003).

### Functional Enrichment Analysis of Targeted DE mRNAs

Metascape (Zhou et al., 2019)^7^ contained many updated functional annotations, such as Kyoto Encyclopedia of Genes and Genomes (KEGG) pathway, canonical pathway, Reactome pathway, Gene Ontology (GO) biological process, and CORUM (the comprehensive resource of mammalian protein complexes). To understand the biological function of targeted DE mRNAs of GSE71332, Metascape was then used with a *p*-value of < 0.01 as the cutoff value. Then the terms with a *p*-value of < 0.01 and a number of genes greater than or equal to 3 were selected as significant terms.

### Transcriptional Regulatory Relationship Analysis of Targeted DE mRNAs

TRRUST (transcriptional regulatory relationships unraveled by sentence-based text-mining)^8^ is a TF-target regulatory interactions database based on the manual curation of Medline abstracts (Han et al., 2015). We then used TRRUST to screen transcription factors related to targeted DE mRNAs and targeted mRNAs and study their transcription regulation relationships.

### Causal Relationship Analysis

DisNor (Lo Surdo et al., 2018)^9^ is a web-based tool that can generate and explore protein interaction networks based on disease genes using Mentha protein interaction data and causal interaction information annotated by SIGNOR. DisNor was used to explore the causal relationships among targeted DE mRNAs.

### Construction and Validation of Nomogram Based on Circulating miRNAs

Logistic regression analysis was then performed with three selected factors by using “survival” R package (Therneau, 2015) to select the best fit model. Then a nomogram was built to predict the risk of RIF patients by using “rms” R package. At the same time, the consistency index (C-index) was calculated to evaluate the model’s ability to distinguish. The consistency of the predicted probability and the actual probability of the model was evaluated by the calibration curves. The predictive performance of the model was evaluated by drawing the receiver operating characteristic (ROC) curve and calculating the area under the curve (AUC) values.

## Results

### Clinical Characteristics of Samples Used in the Study

All data came from samples taken during the window of implantation. The clinical characteristics of the RIF group and the control group in GSE71331 and GSE71332 are listed in Table 1. In total, the mRNA and lncRNA expression profiles of seven RIF samples and five control samples were extracted from GSE71331, and the corresponding miRNA expression profiles were extracted from GSE71332.

**TABLE 1:** Clinical characteristics of GSE71331.

	**RIF**			**CON**			***p*-value**
	**Mean**	***SD***	**n**	**Mean**	***SD***	**n**	
Age (years)	31.5714	4.8599	7	31	3.5355	5	0.7349
Number of failed cycles	5.1429	3.1320	7	0	0	5	0.0032
Number of transferred embryos	12.4286	6.1062	7	2	0	5	<0.000001
Number of high-quality transferred embryos	7.5714	1.5119	7	1.6	0.5477	5	0.0007
Endometrial thickness on the day of LH surge	0.9429	0.1512	7	1.08	0.1095	5	0.9352
The day of sample (post the day of LH surge)	6.5714	0.7868	7	7.2	0.4472	5	0.7096

*RIF, recurrent implantation failure; SD, standard deviation; LH, luteinizing hormone; CON, comment group*

### Selection of Co-expression miRNAs Between Serum and Endometrial Samples and Functional Enrichment Analysis

**FIGURE 1:**
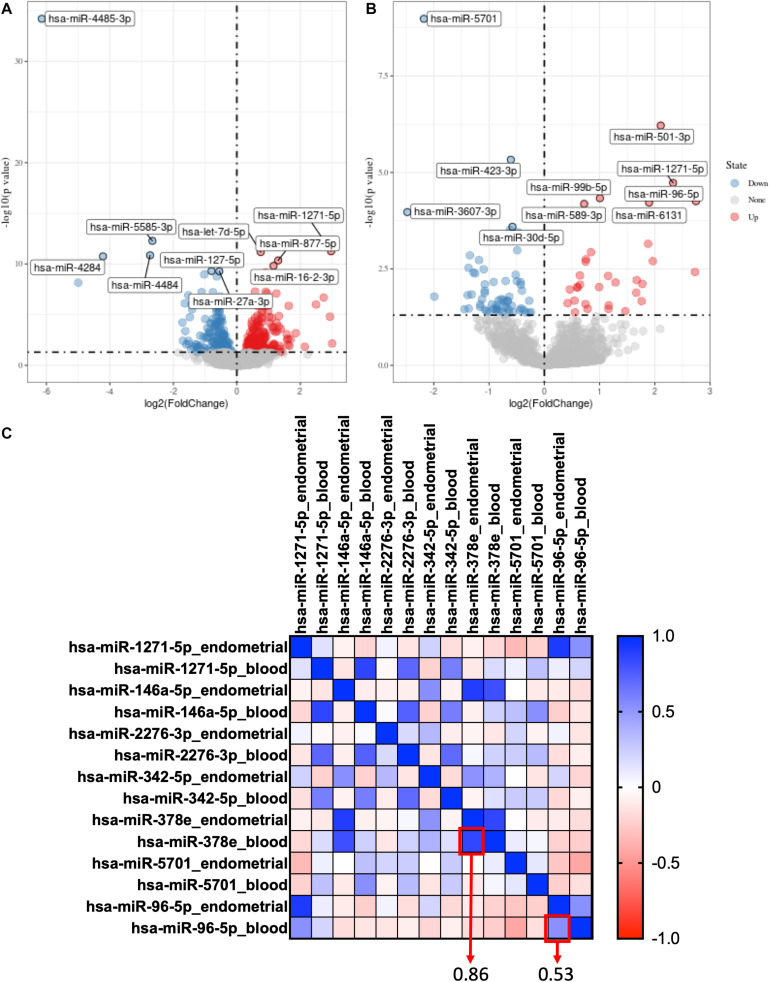
Selection of co-expression miRNAs between serum and endometrial samples. **(A)** The volcano plot of differentially expressed genes in the endometrial sample of GSE108966. **(B)** The volcano plot of differentially expressed genes in the serum sample of GSE108966. **(C)** The heatmap of the co-expression miRNAs between serum and endometrial samples. The numbers inside the boxes stand for correlation coefficient.

For GSE108966, 63 downregulation miRNAs and 45 upregulation miRNAs were selected from endometrial samples by Deseq2 with the following criteria: *p*-value < 0.05 and | log 2-fold change| > 1 (Figure 1A). Similarly, 28 downregulation miRNAs and 22 upregulation miRNAs were selected from serum samples (Figure 1B and Supplementary Table 1).

Hsa-miR-378e and hsa-miR-96-5p were selected as co-expression miRNAs between serum and endometrial samples (Figure 1C).

### Selection of Differentially Expressed Genes

By using “limma” package with *p*-value < 0.05 and | log 2-fold change| > 1, we found that Hsa-miR-378e and hsa-miR-96-5p are also highly expressed in RIF in the profiles of GSE71331 and GSE71332 (Supplementary Table 2).

### Selection of RIF-Related miRNAs by WGCNA

**FIGURE 2:**
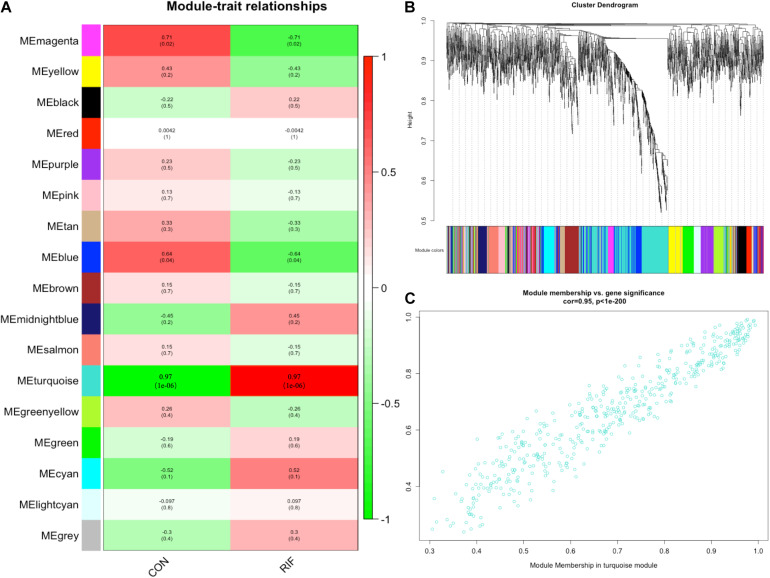
Weighted gene correlation network analysis. **(A)** The relationships between the corresponding modules and clinical phenotypes. **(B)** The modules selected with a minimum module size of 50 for further analysis. **(C)** The module membership in the turquoise module.

A gene co-expression network was then constructed based on the samples of GSE71332 by WGCNA to select the most significant gene modules and genes. This procedure can also help to elucidate the relationship between genes and clinical features. With a soft threshold of β = 7, 16 modules were selected with a minimum module size of 50 for further analysis (Figure 2B).

The overall expression gene level was taken as the MS (module significance) to estimate the relationship between the corresponding modules and clinical phenotypes (Figure 2A). Based on the results, we found that the turquoise module showed the most significant positive correlation with the RIF (cor = 0.97, *p* < 0.0001) (Figure 2C). Therefore, the turquoise module was chosen as the RIF-related module.

Finally, 97 intersection miRNAs between DEG and WGCNA were selected as RIF-related DE miRNAs (Supplementary Table 3).

### Construction of lncRNA–miRNA–mRNA Regulatory Network

**FIGURE 3:**
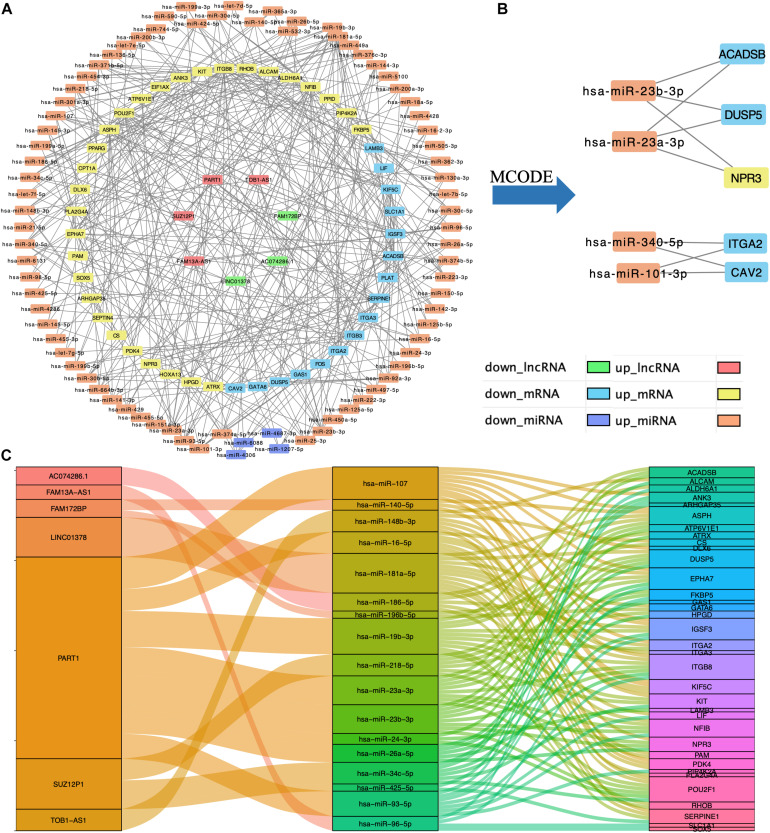
Construction of lncRNA–miRNA–mRNA regulatory network. **(A)** The lncRNA–miRNA–mRNA regulatory network. **(B)** The key module network selected from lncRNA–miRNA–mRNA regulatory network. **(C)** The connection between lncRNAs, miRNAs, and mRNAs.

Based on the interaction of the prediction of three databases (miRDB, miTarBase, and TargetScan) and DE mRNAs, 45 mRNAs were selected for network construction. Similarly, seven lncRNAs were selected. Finally, a lncRNA–miRNA–mRNA regulatory network was constructed based on 80 miRNAs, 45 mRNAs, and 7 lncRNAs by using cytoscape (Figures 3A,C). By using the MCODE app of cytoscape, a key module network was selected (Figure 3B).

### Functional Enrichment Analysis and Causal Relationship Analysis

**FIGURE 4:**
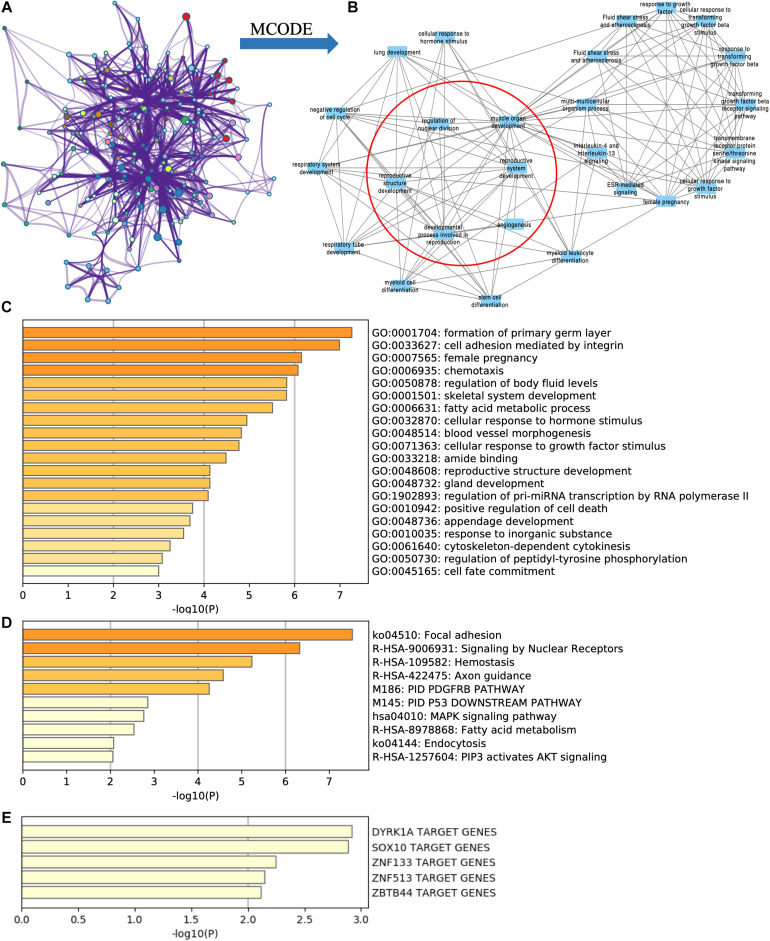
Functional enrichment analysis of targeted DE mRNAs of GSE71331/71332. **(A)** The network of functional terms. **(B)** The key module network selected by MCODE (inside the red circle) and first neighbor node of the key module network (outside the red circle). **(C)** The enriched GO terms of targeted DE mRNAs. **(D)** The enriched pathway of targeted DE mRNAs. **(E)** The transcription factors related to targeted DE mRNAs.

By using Metascape, we found that the targeted DE mRNAs were enriched mainly in terms of cell adhesion mediated by integrin (GO: 0033627), female pregnancy (GO: 0007565), fatty acid metabolic process (GO: 0006631), and reproductive structure development (GO: 0048608) (Figure 4C). The pathways-targeted DE mRNAs were enriched in terms of focal adhesion, signaling by nuclear receptors, MAPK pathway, and PIP3-activated AKT pathway (Figure 4D).

Key modules were selected in the functional network (Figure 4A) by using the MCODE cytoscape app. We found that one of the key modules was closely related with reproduction function (Figure 4B red circle), and their first neighbor nodes are also shown in Figure 4B (outside the red circle).

According to TRRUST database, DYRK1A targeted ACADSB, ANK3, ASPH, HOXA13, ITGA3, and NPR3. SOX10 targeted ACADSB, ANK3, LAMB3, and SOX5 (Figure 4E).

**FIGURE 5:**
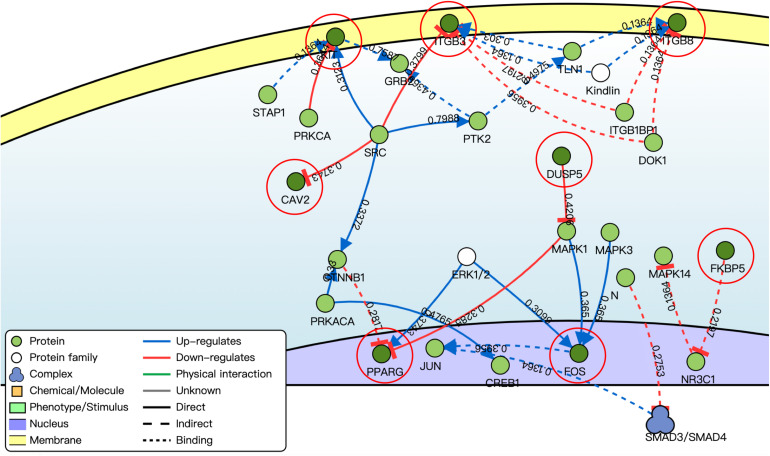
Causal interaction analysis of targeted DE mRNAs of GSE71331/71332 (inside the red circle) and their first neighbor genes (outside the red circle) by using DisNor. The numbers on the line stand for correlation coefficient.

By using the DisNor tool, we screened the first neighboring genes of the targeted DE mRNAs and built a causal relationship network (Figure 5). We found that these genes were closely related to the MAPK signal pathway as we could see in the results of functional enrichment analysis.

### Construction and Validation of Nomogram Based on Circulating miRNAs

**FIGURE 6:**
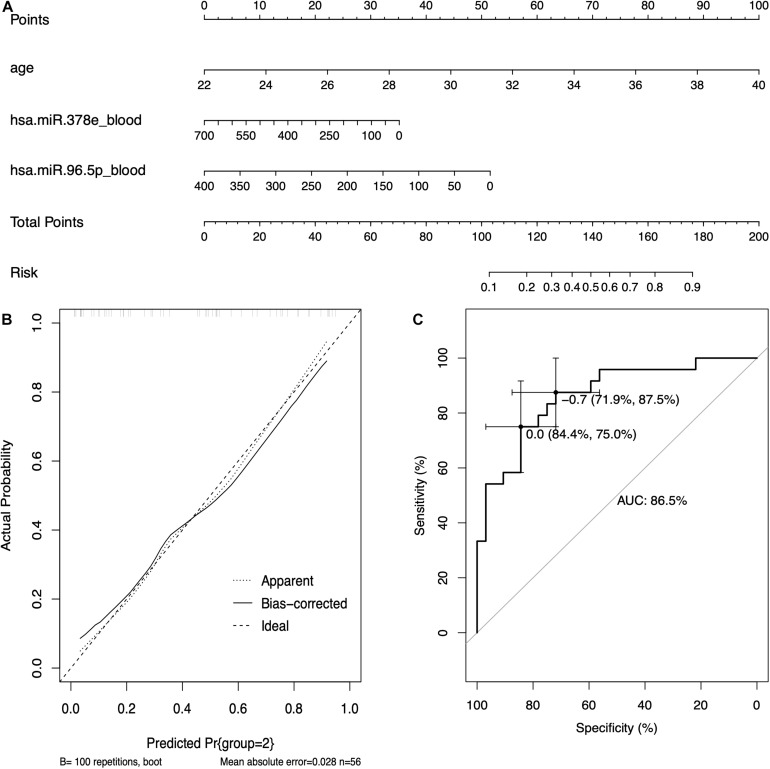
The prediction model based on circulating miRNAs. **(A)** The nomogram of the prediction models based on circulating miRNAs. **(B)** The calibration curves of the prediction model. **(C)** The receiver operating characteristic (ROC) curve of the prediction model.

Based on the results of logistic regression analysis, the best fit models included age, the expression of hsa-miR-96-5p, and the expression of hsa-miR-378e. A nomogram with C-index = 0.865 was established to act as a prediction tool of RIF (Figure 6A), which means that our model has a good ability to distinguish the clinical outcome. The calibration curves in Figure 6B show a good consistency of the predicted probability and the actual probability of the model with mean absolute error of 0.028, mean squared error of 0.00096, and 0.9 quantile of absolute error of 0.044. Figure 6C shows the ROC curve of the model, and the AUC value was 0.865, which means a promising predictive performance.

## Discussion

RIF is an obstacle in the process of assisted reproductive technology (ART). Epigenetic regulation of gene expression played an important role in the development of RIF, and one of the most important parts was miRNA. Several factors, including different miRNAs, were selected as key molecules of regulation of endometrial acceptance and implantation (Kang et al., 2015). However, there was still a lack of research on the molecules that affect endometrial tolerance before implantation and the mechanisms of early dialog between the embryo and the uterus (Kang et al., 2015). Due to the limitations of the sample and detection technology, researchers could only study the known potentially meaningful miRNAs and could not explore them in depth.

In this study, by using WGCNA, we built a lncRNA–miRNA–mRNA regulatory network to analyze the expression and regulation characteristics of miRNAs in the endometrium and the serum. By using the MCODE app in cytoscape, two key modules were selected. We noticed that both hsa-miR-23a and hsa-miR-23b interacted with ACADSB, DUSP5, and NPR3. Fan et al. (2020) reported that the upregulator expression of hsa-miR-23a could suppress hdac2, activate NF-κB, and influence the ability of adhesion, invasion, and proliferation of trophoblasts. Our study showed that hsa-miR-23a played an important role in embryo implantation. At the same time, several studies suggested that hsa-miR-23a and hsa-miR-23b were closely related to the MAPK pathway (Guo et al., 2018; Ma et al., 2019). As many studies reported, the MAPK pathway played an important role in embryo implantation, and it was closely related to the ability of adhesion, invasion, and proliferation of trophoblasts and the procession of endometrium angiogenesis (Baryla et al., 2019; Zhang et al., 2019; Goryszewska et al., 2020). The causal relationship network in Figure 5 shows that DUSP5 downregulates MAPK1 (*R* = 0.42). According to these results, we could make a hypothesis that lncRNA PART1 may act as a sponge of hsa-miR-23a/b to downregulate DUSP5 to promote RIF.

In this study, the results of functional enrichment analysis of miRNAs target genes also support our conclusions. The targeted mRNAs of hsa-miR-96-5p were mainly enriched in terms of cellular response to organonitrogen compound, negative regulation of cell differentiation, regulation of protein serine/threonine kinase activity, apoptosis pathway, and the MAPK signaling pathway.

Currently, almost all tests for endometrial function in RIF patients are based on endometrial biopsies. Such an inspection operation had a potential impact on the uterine cavity environment. In this study, we developed a non-invasive RIF diagnostic scoring model to assist in the diagnosis and treatment of RIF patients, and it showed better predictability and accuracy. As far as we know, this was the first RIF predictive scoring model based on circulating miRNA. Clinical trials of models will also be conducted soon. For this study, there were still some shortcomings, such as a lack of adequate laboratory tests to verify the mechanism. We are already starting relevant clinical studies.

## Conclusion

In this study, we built a circulating miRNA-based prediction and provided a new non-invasive inspection method. We also found that these two miRNAs played an important role in the progress of RIF and found that lncRNA PART1 may act as a sponge of hsa-miR-23a/b to downregulate DUSP5 to promote RIF.

## Data Availability Statement

The datasets presented in this study can be found in online repositories. The names of the repository/repositories and accession number(s) can be found in the article/Supplementary Material.

## Author Contributions

CF and PC carried out the study, coordinated the study, participated in the design, and reviewed the manuscript. PC analyzed and interpreted the data. YG and TL drafted the manuscript. LJ and YW collected and analyzed the data. All authors read and approved the final manuscript.

## Conflict of Interest

The authors declare that the research was conducted in the absence of any commercial or financial relationships that could be construed as a potential conflict of interest.

## Publisher’s Note

All claims expressed in this article are solely those of the authors and do not necessarily represent those of their affiliated organizations, or those of the publisher, the editors and the reviewers. Any product that may be evaluated in this article, or claim that may be made by its manufacturer, is not guaranteed or endorsed by the publisher.

## Footnotes

1 http://www.ncbi.nlm.nih.gov/geo

2 http://mirdb.org/miRDB/

3 http://mirtarbase.mbc.nctu.edu.tw/

4 http://targetscan.org/

5 http://bigdata.ibp.ac.cn/npinter4/

6 https://cytoscape.org/

7 http://metascape.org

8 http://www.grnpedia.org/trrust

9 https://disnor.uniroma2.it/

## Supplementary Material

Supplementary Table 1 The co-expression miRNAs between serum and endometrial samples.

Supplementary Table 2 The differentially expressed genes (DEGs) list of GSE71331/71332.

Supplementary Table 3 The intersection miRNAs between DEG and WGCNA in endometrium of RIF patients.

## Abbreviations

RIF: recurrent implantation failure
ART: assisted reproductive technology
ceRNA: competing endogenous RNA
WOI: window of implantation
WGCNA: weighted correlation network analysis.
